# Supplementary material for: Targeted temperature management at 33°C or 36℃ induces equivalent myocardial protection by inhibiting HMGB1 release in myocardial ischemia/reperfusion injury
Source: PLoS One. 2021 Jan 27;16(1):e0246066. doi: 10.1371/journal.pone.0246066 (PMC7840046; doi:10.1371/journal.pone.0246066)
Supplement: S1 Table — (DOCX) [file pone.0246066.s001.docx]

**S1 Table. Sequences**

| Name | Sequence |
| --- | --- |
| TNF-α | Forward: TAG CAA ACC ACC AAG CAG AG  Reverse: GAG AACC TGG GAG TAG ATA AGG |
| IL-1β | Forward: CTA TGG CAA CTG TCC CTG AA  Reverse: GGC TTG GAA GCA ATC CTT AAT C |
| IL-6 | Forward: GAA GTT AGA GTC ACA GAA GGA GTG  Reverse: GTT TGC CGA GTA GAC CTC ATA G |
| GAPDH | Forward: ACT CCC ATT CTT CCA CCT TTG  Reverse: CCC TGT TGC TGT AGC CAT ATT |
| HMGB1 | Forward: ATT GCT GCC TAC AGA GCT AAA  Reverse: GTC GTC TTC CTC TTC CTT CTT T |
