# Supplementary material for: Targeted temperature management at 33°C or 36℃ induces equivalent myocardial protection by inhibiting HMGB1 release in myocardial ischemia/reperfusion injury
Source: PLoS One. 2021 Jan 27;16(1):e0246066. doi: 10.1371/journal.pone.0246066 (PMC7840046; doi:10.1371/journal.pone.0246066)
Supplement: Attachment — Submitted filename: Response to reviewers comment plos one TTM.docx [file pone.0246066.s003.docx]

**Review Comments to the Author**

Reviewer #2: Comments to Authors after paper revision
The Authors did their best to replay to all my comments on their article and I’m quite satisfied of their revision: new figures showed better quality, the results conserning missing experimental groups were provided, they performed ELISA assay for HMGB1 in serum, and I appreciated their care in clarifying my doubts about time of reperfusion and timing of HMGB1 release. I think that the present article had considerably improve its quality after the revision. For this reason, I agree for the publication, even if, in my opinion is still compulsory to better clarify the question of Glycyrrhizin.

I understood the impossibility for the Authors to conduct experiments using pharmacological agents that induce HMGB1 release, but I think that is still not sufficiently clear in the paper that the second part of experiments (the part with Glycyrrhizin) did not provide the direct demonstration that the cardioprotective effect of TTM is mediated by the blocking of HMGB1 release.

In fact, in my opinion, the blocking of HMGB1 release could be just a consequence and not be directly dependent on TTM. When the Authors compared Glycyrrhizin treatment with TTM, is just a speculation: the two treatments have the same final effect (myocardial protection during I/R), and present the same phenomenon (inhibition of HMGB1 release), but the mechanisms might be completely different. Glycyrrhizin impedes for sure HMGB1 release from cardiomyocytes because is a pharmacological inhibitor, TTM, on the contrary, might potentially act on some other factors that merge on the blocking of HMGB1 release. The effects are similar, but the mechanisms just could be. And the experiments with Glycyrrhizin did not provide any evidence on it; they only showed similarity between the two treatments and the Authors are just speculating, not demonstrating anything on this point. This has to be clear in order for the publication of the present paper.

**Response to Reviewer’s Comment**

**Response:** Thank you for your helpful comment; we agree with it. Per your comment, we also think that the inhibition of HMGB1 release may be one of the mechanisms underlying the cardioprotective action of TTM and may result from several protective effects of TTM after I/R injury. Although the experiments involving glycyrrhizin and neutralizing antibodies only demonstrated that the extracellular release of HMGB1 is a key factor in the cardiac damage after I/R injury, there is no evidence that the blockade of the HMGB1 release is direct mechanism of the TTM-induced cardiac protection after I/R injury. We have mentioned this point in the Discussion section of the revised manuscript.

Discussion

We demonstrated that the serum HMGB1 level was lower in the TTM group than in the group without TTM, revealing a correlation between TTM and HMGB1. However, our study lacks data regarding the link between HMGB1 and I/R injury and on the direct mechanisms underlying this association. In the case of the AMI animal model, we could not confirm the relationship between HMGB1 and I/R injury because HMGB1-neutralizing antibodies could not be injected locally. Instead, we indirectly revealed the association of HMGB1 with I/R injury by using glycyrrhizin as a pharmacological HMGB1 inhibitor and via experiments using our middle cerebral artery occlusion model [24, 26]. However, these analyses with glycyrrhizin and neutralizing antibodies only demonstrated that the extracellular release of HMGB1 is a key factor in the cardiac damage after I/R injury. There is no evidence that the blockade of the HMGB1 release is direct mechanism of the TTM-induced cardiac protection after I/R injury. Further studies are needed to identify the direct mechanisms underlying, and the link between, HMGB1 release and the action of TTM after I/R injury. In addition, Liu et al. have identified several classes of agents that potently induce the nucleo-cytoplasmic relocation and subsequent cellular release of HMGB1 [45]. To clarify whether TTM can attenuate the inflammatory response in peri-infarct regions by inhibiting the extracellular release of HMGB1 after I/R injury, further studies are needed to verify the ischemic damage by using pharmacological agents that induce HMGB1 release during TTM after I/R injury in the AMI model.
